# COVID-19 severity in relation to sociodemographics and vitamin D use

**DOI:** 10.1515/med-2021-0273

**Published:** 2021-04-08

**Authors:** Darya Saeed Abdulateef, Heshu Sulaiman Rahman, Jamal Mahmood Salih, Sangar Mahmoud Osman, Trifa Abdalla Mahmood, Shirwan Hama Salih Omer, Rana Adnan Ahmed

**Affiliations:** Department of Physiology, College of Medicine, University of Sulaimani, New–Street-27, Zone 209, P. O. Box: 334, Kurdistan Region, Sulaymaniyah, Iraq

**Keywords:** COVID-19, symptoms severity, vitamin D, smoking, online survey

## Abstract

Most COVID-19 cases are treated as outpatients, while the majority of studies on COVID-19 focus on inpatients. Little is known about the self-reporting and self-rating of the disease’s symptoms, and the associations of prophylactic use of dietary supplements with COVID-19 severity have not been addressed. The aims of this study are to evaluate COVID-19 severity and to relate them to sociodemographic characteristics and prophylactic dietary supplements. An observational patient-based study conducted through an online questionnaire on recovered COVID-19 patients. The patients were assessed for several severity parameters, sociodemographic parameters, and prophylactic dietary supplement use. A total of 428 patients were evaluated. Age and presence of comorbidities had positive associations with the severity parameters. The severe infection group had the highest proportion of patients stressed about COVID-19 (*P* < 0.05). Cigarette, but not hookah, smoking was significantly associated with less severe symptoms. Vitamin D negatively predicted disease severity (*P* < 0.05). In conclusion, stress, age, and presence of comorbidities were the most important positive predictors of COVID-19 severity, while prophylactic vitamin D use and smoking were significant negative predictors. The use of protective measures and other prophylactic dietary supplements was not significantly associated with symptom severity.

## 1 Introduction

Several factors such as sociodemographic factors, the use of protective measures, and the use of prophylactic supplements have been linked to COVID-19 infection and severity. Age, body mass index (BMI), and comorbidities are among the most important demographic parameters; other factors such as sex, smoking status, place of residence, housing condition, nature of employment, and concern about COVID-19 are also believed to be associated with this infection and its severity. Regarding age, a slightly lower infection rate in young people has been reported in addition to a lower death rate (less than 1%) [1,2]. Meanwhile, BMI is positively associated with COVID-19 severity [3,4]. The COVID-19 rate has been reported to be higher in males than in females [5]. Pre-existing medical conditions have been linked with poor outcomes of COVID-19 infection [6,7,8].

Regarding smoking status in relation to the COVID-19 rate and severity, there is disagreement between the reported data [9,10,11,12,13], in which some studies have reported no association of active smoking with COVID-19 severity [10], whereas others have reported that active smokers are associated with an increased severity rating and worse outcomes [11,12,14]. Hookah is an important source of SARS‐CoV‐2 transmission [15]. Regarding alcohol, misuse of alcohol affects the body’s immune function and increases an individual’s risk of viral infection [16,17,18]. Chronic and excessive alcohol consumption affect the lung epithelial cells, increasing the risk of severe acute respiratory distress syndrome (ARDS) [18]. The prevalence of smoking cigarette and hookah and alcohol drinking is low in Iraq, with a male vs female prevalence of 40.4 vs 2.9% and 12.3 vs 0.4% and 14.7 vs 1.2%, respectively [19].

Concern and stress about COVID-19 are a significant problem that has emerged during this pandemic [20]. Social isolation, staying home, feeling lonely, and getting stressed about COVID-19 infection make people more vulnerable to mental stress, sleep disturbances, eating problems, and comorbidity progression [20,21].

To some extent, an individual’s place of residence (urban, suburban, or rural) has an effect on the transmission and spread of COVID-19 [22,2]. The more crowds and industries a city has, the more frequently people will come into contact with one another [23,24]. Housing conditions are also important during the COVID-19 pandemic because of the need for home isolation, which requires a separate bedroom, restroom, kitchen, and plumbing system [25,26]. Places with a higher percentage of poor housing conditions have higher rates of infection and fatality associated with COVID-19 [27,28]. It has been more common for COVID-19 infection to be transmitted more frequently within households than what was observed with Middle East respiratory syndrome coronavirus [29]. Moreover, SARS‐CoV‐2 transmissions from person to person within the family cluster have primarily been from pre-symptomatic people [30], and more than half of all SARS‐CoV‐2 transmissions has been from family members [31].

Furthermore, an individual’s job, regular daily work, and hours spent outside have an effect on COVID-19 infection in that a particular job can put an individual at greater risk [32].

The use of protective measures such as social distancing, wearing a face mask, washing hands with soap and water, and disinfecting hands regularly is highly encouraged by authorities during COVID-19 outbreaks [33,34,35], as the transmission of this infection is high and rapid [36]. In addition, a large number of asymptomatic COVID-19 patients who have not adopted protective measures play a major role in the high transmission of the virus [37]. Hand hygiene is an important protective measure to decrease the severity and death rate of the disease [38].

Along with the use of protective measures to combat COVID-19, a number of prophylactic dietary supplements and drugs have been advised to enhance the immune system or decrease viral replication. A recently published review stated that micronutrients support the immune system and may reduce the risk of infection [39]. Among them, vitamin D is believed to suppress viral replication and affect the pro- and anti-inflammatory cytokine concentration, which may affect the mucosal lining of the lungs and cause the development of pneumonia [40]. In addition, vitamin D deficiency has been linked to an increased risk of ARDS and acute respiratory tract infection [41,42].

Similarly, vitamin C (ascorbic acid) could play a role in the prevention and treatment of viral infections by different mechanisms such as scavenging free oxygen radicals, reducing pro-inflammatory cytokines, and enhancing the antimicrobial ability of certain cells [43,44]. A randomized clinical trial showed that intravenous vitamin C reduced the mortality but did not alter the disease severity in patients with ARDS or sepsis [45,46]. Meta-analyses have shown that vitamin C modestly shortens the duration of common cold caused by human coronavirus [47], the duration of ICU stay [48], and the duration of mechanical ventilation [49].

The trace mineral zinc is hypothesized to prevent viral attachment to the nasopharyngeal mucosa and inhibit viral replication. Different doses of zinc have been used in registered clinical trials for COVID-19, with a maximum dose of 50 mg twice daily [50].

To the best of our knowledge, no COVID-19 patient-based study has been reported to correlate the usage of dietary supplements with symptom severity, and only incomplete data are available on the relation of several sociodemographic parameters with the severity rating, symptom score, and frequency of protective measure use among COVID-19 patients. Moreover, there is disagreement over COVID-19 symptom severity and smoking status. Therefore, the aims of this study are to assess the frequency of dietary supplements used by the studied population and to analyse the association between COVID-19 severity and the use of these prophylactic measures and dietary supplements in conjunction with several socio-demographic parameters. Moreover, this research assessed the rate of the patients’ symptoms, complications, hospitalization, hospital visit, medical consultation, oxygen therapy, plasma transfusion, and sleep disturbances.

## 2 Materials and methods

### 2.1 Study design and participants

This was an observational study of a convenience sample of patients who recovered from COVID-19, and this study was conducted in the Sulaymaniyah Governorate (Iraq) between July and August 2020 through an online patient-based survey via Google Forms in three different languages (Kurdish, Arabic, and English). The required sample size was calculated according to the expected prevalence of COVID-19 in the Sulaymaniyah Governorate (Iraq), which indicated the need for at least 345 participants with the degree of precision set at 10%. Participation was anonymous and voluntary. The participants were assured of the confidentiality of the survey and that no one could access their information. The participants’ online questionnaire responses were accepted for a 1-week period from 23 to 31 July 2020. On 1st August, the online forms were closed and no longer accepted responses.

Three survey links were shared through social media, including a page dedicated to give advice and health information on COVID-19 infection and also to encourage patients to donate plasma, a page for medical doctors and health staff, and a page for Ministry of Education schoolteachers. In addition, emails were sent out via the deanery of the College of Medicine, University of Sulaimani, to all college staff. The staff were asked to share the survey link with their family members, relatives, and friends who had contracted COVID-19.


**Ethics statement:** The ethical approval for this study was obtained from the Ethical and Scientific Committee of Physiology Department and the Ethical Committee of the College of Medicine, University of Sulaimani, with reference number 17, on 18 July 2020, with meeting number 141.

### 2.2 Inclusion criteria

Patients belonging to the Sulaymaniyah Governorate (Iraq) who had previously been infected with COVID-19 and cured were the study participants. The inclusion criteria included patients confirmed to have had COVID-19 infection based on one or more of the following positive tests: RT-PCR of the nasopharyngeal swab, high-resolution CT (HRCT) of the chest or typical signs and symptoms of COVID-19, chest X-ray (CXR), and serological investigation. The patients with COVID-19 symptoms were retrospectively characterized as to whether they met the case definition approved by the Council of State and Territorial Epidemiologists [51]. The patients had to meet one or more of these two groups of criteria: (a) cough, shortness of breath (SOB), or dyspnoea and/or (b) at least two of the following signs: fever, rigour, myalgia, headache, sore throat, dysgeusia, and anosmia.

The patients had to have completely healed, that is, to be symptom free for 2 weeks or for 4 weeks since their diagnosis.

### 2.3 Exclusion criteria

Survey respondents who failed to meet the study eligibility criteria were excluded from the study, such as participants who reported to have been diagnosed on the basis of only one of the following: serology, CXR, or symptoms with the absence of typical COVID-19 symptoms upon checking their symptom profile (i.e. they did not meet the previously mentioned case definition of Council of State and Territorial Epidemiologists).

### 2.4 Questionnaire design

The study started with the preparation of a proper and detailed questionnaire designed by the authors using Google Forms; the survey was piloted among the research team members who filled out and reviewed the questionnaire, and it was accordingly edited before being distributed among the study participants.

The responses of the pilot study were not included in the main survey data. When the validation process was complete, the survey was translated into Kurdish and Arabic by three native speaker physicians, who are members of the research team, and edited by two other members before distribution. Translations were made available in three languages: Kurdish (mother language), Arabic, and English. The last two languages refer to the second and third spoken languages among the Sulaymaniyah citizenry. It was an easy and understandable form to be filled in by the participants.

The survey started with a description of the eligibility to participate, followed by the purpose of the study. It was made clear to the participants that they could withdraw at any time. The first question was about the participants’ consent to contribute to the survey and details about their socio-demographic characteristics.

The questionnaire contained a total of 39 questions about the following topics:

- **(a) General and personal patient characteristics**
  This included five questions about general patient characteristics such as age, gender, height, weight, and marital status, and three questions about personal characteristics such as smoking status (whether they smoke or not), type of smoking (whether cigarette, hookah, or both), number of cigarettes smoked per day [or amount of hookah smoked (in hours/week)], alcohol drinking status (whether they drink alcohol or not), and exercise (whether they exercise daily, weekly, or rarely).
- **(b) Place of residence, work, and risk**
  This included three questions about the participants’ residency, housing conditions, and household and five questions about their work (whether they are unemployed, self-employed, health staff, or an employee other than the health staff), regular daily work, time spent outdoors, close contact with COVID-19 patients, concern about COVID-19 infection before becoming infected, and chronic diseases/comorbidities (whether they have hypertension/heart diseases, diabetes mellitus, autoimmune disease or organ transplant, respiratory diseases, thyroid diseases, cancer, and rheumatological diseases).
- **(c) The use of protective measures**
  This included questions on whether the participants strictly and regularly implemented common protective measures (i.e. social distancing, face mask wearing, handwashing, and staying away from infected or suspected patients) before they were infected with COVID-19.
- **(d) Prophylactic drug and dietary supplements**
  This included six questions to obtain information about their use of prophylactic dietary supplements (vitamin D, vitamin C, and zinc), their dosages, and the duration of use (the number of weeks of use before COVID-19 diagnosis).
- **(e) COVID-19 severity and symptom profile**

This was the main part of the questionnaire and included 11 questions on whether the participants had experienced any symptoms during their COVID-19 infection (symptomatic) or whether they were asymptomatic. If they selected “yes” for symptomatic, they were directed to another section on COVID-19 severity. The COVID-19 severity questions comprised two parts, which are outlined below.

#### 2.4.1 Severity parameters (I)

This section included five questions (Table 2). The first question was on rating the symptom/disease severity in general (severity rating), followed by a question on rating ten individually listed symptoms. The listed symptoms were malaise, fever, rigour and/or sweating, myalgia, headache, sore throat, shortness of breath, dry cough, loss of taste and/or smell, chest pain (pleurisy), and gastrointestinal symptoms (abdominal pain, nausea, and vomiting, and/or diarrhoea). The ratings involved a visual rating scale from 0 to 5, derived from a visual analogue scale [52]: no symptoms (0), very mild (1), mild (2), intermediate (3), severe (4), and very severe (5). The no-symptom option was not included in the general severity rating, because only the symptomatic patients were asked to indicate the severity of their disease condition. The patients were separated according to their rating of severity into mild-moderate (<4) and severe groups (≥4). The symptom score was calculated by adding all rating symptoms, and a score out of 50 was calculated as the symptom score for that patient: five was the highest symptom score for each symptom, meaning that 50 was the highest possible symptom score for the ten listed symptoms.

In this part, the participants were also asked two separate questions: their highest recorded body temperature and lowest recorded SpO_2_ during the time of infection (if available), followed by a question about the symptom duration of their disease (in days).

#### 2.4.2 Severity parameters (II)

In this section, the participants were asked whether they were hospitalized and asked six questions (yes/no) as to whether the disease made them seek any of the following: visiting a hospital, consulting a medical specialist, using medication, using oxygen therapy, receiving plasma from patients who had recovered from COVID-19, and using medication to help them sleep.

- **(a) Treatment and sequelae**

This section covered 11 choices for the treatment/medication the participants received during their infection and 7 choices for the sequelae/complications (malaise, memory, and attention defect; renal problem; respiratory problem; gastrointestinal tract (GIT) problem; and none) that they acquired after their recovery.

### 2.5 Term definitions and variable grouping

The participants were divided according to their age into four groups: paediatric (<18 years), adult (18–39 years), middle aged (40–64 years), and old age (≥65 years).

According to BMI, the participants were divided into four groups: underweight (<18.5 kg/m^2^), normal weight (18.5–24.9 kg/m^2^), overweight (25–29.9 kg/m^2^), and obese (≥30 kg/m^2^).

Regarding the severity rating, the symptomatic patients were divided into two groups: the mild-moderate group (rating <4) and the severe group (rating ≥4). Fever with or without rigour/sweating, coughing, and SOB were regarded as typical symptoms [53].

### 2.6 Statistical analysis

The patients’ responses were transferred into a single SPSS programme; IBM SPSS version 22 (Chicago, IL, USA); and the variables were coded and analysed for socio-demographics, use of protective measures, and dietary supplements. A normality test was performed using the Kolmogorov–Smirnov test, and all the continuous variables were non-parametric. A descriptive statistical analysis was done to find the median of each variable within the interquartile range (IQR).

Numerical variables were shown as frequency and percentages. A chi-square test was applied for all categorical variables, and COVID-19 severity was compared between different age groups, BMI groups, the presence or absence of chronic diseases, and the use of dietary supplements. The descriptive parameters, such as the variables in COVID-19 severity parameters (I), were compared between groups based on age, BMI, dietary supplement use, and with other parameters using the Mann–Whitney *U* test and Kruskal–Wallis *H* test. Spearman’s correlation was used to find a linear correlation between general socio-demographics and COVID-19 severity parameters. A linear regression analysis was performed to determine the degree of prediction between parameters of significant correlation. *P* ≤ 0.05 was regarded as significant. Multiple linear regression analysis was performed to quantify the correlation between patient characteristics and severity parameters as outcome. Stacked bar charts, box plots, and scatter plots were used to demonstrate significant data.

## 3 Results

A total of 455 participants consented to fill in the form. Among them, 27 were excluded because of failure to meet the eligibility criteria and the case definition. After enrolment, 428 patients were entered into the final analysis of the study. The participants’ age ranged from 15 to 80 years, with a median age of 33 years (IQR = 18 years).

### 3.1 General characteristics and socio-demographic parameters

The general and socio-demographic characteristics of the studied participants are shown in Table 1.

**Table 1:** General characteristics and socio-demographic parameters of the studied participants

Parameter		*N* (%)	Total
**Median age (IQR, min–max) = 33 (18, 15–80) years**			428
Age groups	<18	8 (1.9)	
	18–39	286 (66.8)	
	40–64	128 (29.9)	
	>65	6 (1.4)	
**Median BMI (IQR, min–max) = 25.75 (5.62, 15.74–62.1) kg/m^2^**			413
BMI groups	Underweight	15 (3.6)	
	Normal weight	146 (35.4)	
	Overweight	179 (43.3)	
	Obese	73 (17.7)	
Sex	Male	190 (44.4)	428
	Female	238 (55.6)	
Marital status	Single	164 (38.7)	424
	Married	255 (60.1)	
	Divorced	5 (1.2)	
**Family member median (IQR, min–max) = 5 (2, 1–14)**			428
Place of living	Urban	388 (90.9)	427
	Suburban	32 (7.5)	
	Rural	7 (1.6)	
Housing condition	Good	324 (75.7)	428
	Intermediate	101 (23.6)	
	Poor	3 (0.7)	
Job	No work	119 (27.8)	428
	Self-employed (free work)	55 (12.9)	
	Health staff	126 (29.4)	
	An employee	128 (29.9)	
Regular daily work	No	214 (50.0)	428
	Yes	214 (50.0)	
Time spent out (hours)	<1	135 (31.5)	428
	1–3	86 (20.1)	
	4–6	85 (19.9)	
	6	122 (28.5)	
Presence of a close contact	No known contact	181 (42.3)	428
	Yes, family member	173 (40.4)	
	Yes, friends	22 (5.1)	
	Yes, colleague at work	52 (12.1)	
Stress/concern about the COVID-19	Not concerned	108 (25.2)	428
	To slight extent	147 (34.3)	
	Intermediate	112 (26.2)	
	To great extent	61 (14.3)	
Presence of chronic diseases	No	378 (88.3)	428
	Yes	50 (11.7)	
	Hypertension and heart diseases	31 (62.0)	
	Diabetes mellitus	8 (16.0)	
	Autoimmune disease or organ transplant	4 (8.0)	
	Respiratory diseases	3 (6.0)	
	Thyroid diseases	3 (6.0)	
	Cancer	3 (6.0)	
	Rheumatological diseases	1 (2.0)	
Smoking	No	367 (85.7)	428
	Yes	61 (14.3)	
Smoking type	None	367 (85.8)	423
	Cigarette	24 (5.7)	
	Hookah	20 (4.7)	
	Both	12 (2.8)	
Number of cigarette smoked/day	<10	14 (21.2)	66
	10–20	15 (22.7)	
	>20	7 (10.6)	
	No	30 (45.5)	
Amount of hookah smoked (hours/week)	<3	25 (37.9)	66
	3–4.5	2 (3.0)	
	>4.5	5 (7.6)	
	No	34 (51.5)	
Drinking alcohol	No	391 (91.4)	428
	Yes	37 (8.6)	
Exercise	Rarely	295 (68.9)	428
	Weekly	90 (21.0)	
	Daily	43 (10.0)	
Sequelae/complication after COVID-19	Malaise	231 (56.8)	
	Memory and attention defect	89 (21.9)	
	Renal problem	37 (9.1)	
	Respiratory problem	88 (21.6)	
	GIT problem	35 (8.6)	
	None	144 (35.4)	

### 3.2 Parameters of COVID-19 severity

Among the study participants, 406 (94.9%) were symptomatic. After analysing the parameters, the severity of COVID-19 infection in the symptomatic patients is given below.

#### 3.2.1 Severity parameters (I)

The median (range) of each severity parameter is demonstrated in Table 2. According to the general severity rating, 285 (70%) participants were within the mild-moderate group, and 122 (30%) were in the severe group.

**Table 2:** Parameters of COVID-19 severity I

Parameter	Total no.	Total	Mild-moderate	Severe	*P* value
		Median (IQR)	Median (IQR)	Median (IQR)	
		Min–max	Min–max	Min–max	
1. Severity rating	407	3 (2)	3 (1)	4 (1)	<0.001
		1–5	1–3	4–5	
2. Symptom score	407	24 (14)	22 (11)	32 (13)	<0.001
		2–47	2–41	7–47	
3. Highest recorded body temperature (°C)	237	38 (1.5)	38 (1.3)	38.9 (1.2)	<0.001
		35–43	35–43	36.7–43	
4. Lowest recorded SpO_2_ (%)	225	94 (7)	94 (4)	90 (13)	<0.001
		50–99	60–98	50–99	
5. Symptom duration (days)	395	14 (10)	14 (9)	18 (9)	<0.001
		1–40	1–37	3–40	

#### 3.2.2 Severity parameters (II)

The frequency (percentage) of each severity parameter is summarized in Table 3.

**Table 3:** Parameters of COVID-19 severity II

Seeking condition	Yes	No
	*N* (%)	*N* (%)
Hospitalization	30 (7.0)	398 (93.0)
Hospital visit	179 (41.8)	249 (58.2)
Consult a medical specialist	303 (70.8)	125 (29.2)
Use of treatment (medication)	297 (69.4)	131 (30.6)
O_2_ therapy	44 (10.3)	384 (89.7)
Received plasma	19 (4.4)	409 (95.6)
Affect your sleep	147 (34.3)	281 (65.7)

The comparison of all the parameters of severity (I and II) between the mild-moderate and severe groups of patients is presented in Table 2 and supplemental file (Figure S1).

### 3.3 Symptom profile and rating of each separate symptom (symptom score)

**Figure 1:**
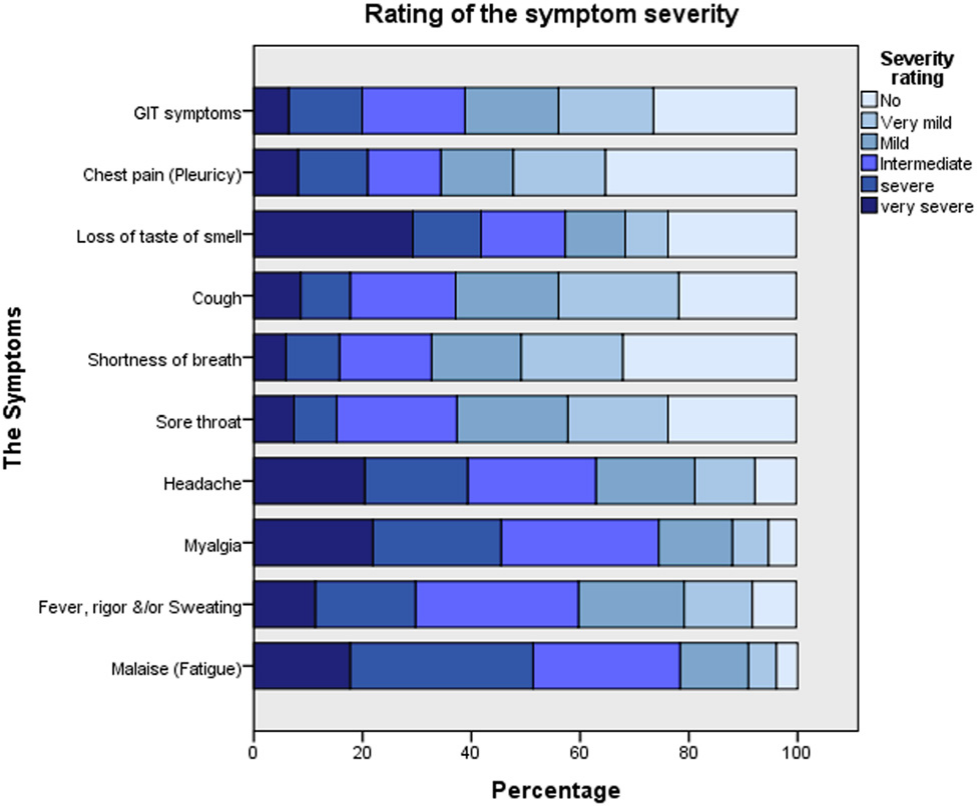
Percentage of symptom severity rating by the patients according to each separate symptom or symptom groups.

The most common symptoms among the symptomatic patients were malaise (96.1%), myalgia (94.8%), and headache (92.4%), followed by fever and/or rigour and sweating (91.9%). Moreover, 78.4% of the patients reported having a cough, and 76.4% experienced loss of taste and/or smell with a sore throat (Figure 1). The symptoms that presented with the highest degree of severity among the patients were loss of taste and/or smell in 29.2%, followed by myalgia and headache in 21.9 and 20.4%, respectively. Among the symptomatic patients, 54.8% (223) had all three typical symptoms (fever with or without rigour/sweating, coughing, and SOB).

Regarding sequelae, 144 (35.4% of the symptomatic) patients had no sequelae, while the others had one complication (35.9%), two complications (18.2%), or more than two complications (10.5%). The most common sequelae among participants were malaise (56.8%) and respiratory problems (21.6%).

### 3.4 Relation of socio-demographic parameters with COVID-19 severity rating

The multiple logistic regression analysis of COVID-19 severity predictors (with severity rating as an outcome variable) was performed and demonstrated in Table 4. Concerns about COVID-19, age, and chronic disease were the most significant positive predictors (standardized *β* of 0.23, 0.01, and 0.15; *P* < 0.001, 0.01, and 0.04, respectively), while smoking negatively predicted symptom severity (standardized *β* of −0.14; *P* = 0.01).

**Table 4:** Socio-demographic parameters and prophylactic dietary supplements as predictors of COVID-19 severity through multiple linear regression analysis

Model	Unstandardized coefficients		Standardized coefficients	*P* value	95.0% Confidence interval	
	B	S.E			Lower bound	Upper bound
Sex	0.02	0.13	0.009	0.88	−0.24	0.28
**Age**	0.01	0.005	0.15	**0.01**	0.003	0.02
BMI	−0.006	0.01	−0.03	0.56	−0.03	0.01
**Smoking**	−0.43	0.17	−0.14	**0.01**	−0.75	−0.10
Drinking alcohol	0.17	0.20	0.04	0.41	−0.23	0.56
Exercise	−0.02	0.08	−0.009	0.85	−0.17	0.14
Place of living	0.27	0.15	0.09	0.08	−0.03	0.57
Number of family members	−0.02	0.03	−0.03	0.60	−0.07	0.04
Housing condition	0.02	0.13	0.007	0.89	−0.23	0.26
Job	−0.02	0.05	−0.02	0.71	−0.12	0.08
Regular daily work outside home	0.25	0.17	0.12	0.13	−0.07	0.57
Time spent out (hours)	−0.01	0.07	−0.01	0.89	−0.15	0.13
**Degree of concern**	0.24	0.05	0.23	**<0.001**	0.14	0.35
Close contact	−0.02	0.11	−0.008	0.87	−0.23	0.19
**Chronic diseases**	0.36	0.17	0.11	**0.04**	0.02	0.70
Vitamin D use	−0.38	0.17	−0.16	**0.03**	−0.71	−0.05
Vitamin C use	0.13	0.19	0.06	0.49	−0.24	0.50
Zinc use	0.004	0.19	0.002	0.98	−0.37	0.37

Note: The bold values refer to the parameters and P values with significant results among others.

### 3.5 Relation of socio-demographic parameters with other severity parameters

**Figure 2:**
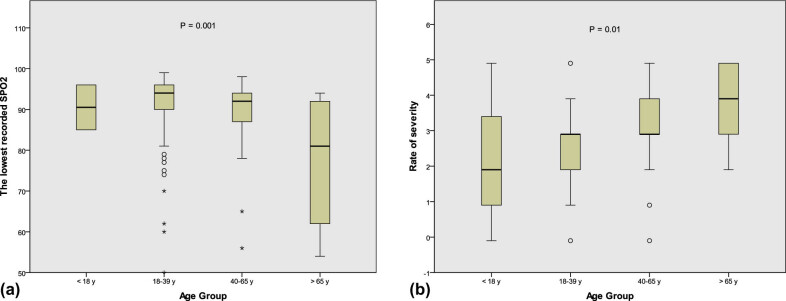
Comparison of severity rating (a) and lowest recorded SpO_2_ (b) between the different age groups.

When age was related with the other severity parameters, it was positively associated with the symptom duration (*r* = 0.15; *P* = 0.003) and negatively correlated with SpO_2_ (*r* = −0.19; *P* = 0.004). The severity of COVID-19 was compared between the four age groups, and significant results were found (Figure 2a and b).

**Figure 3:**
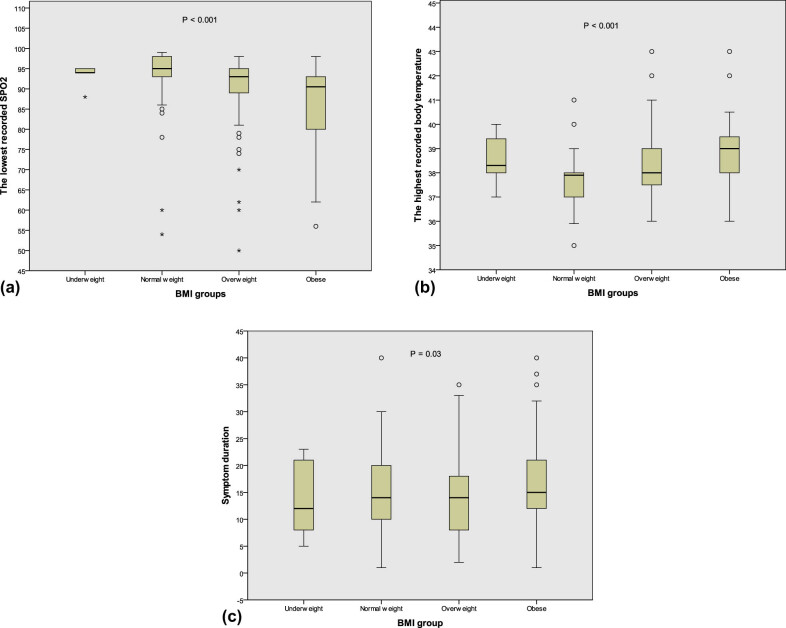
Comparison of the lowest recorded SpO_2_ (a), highest recorded body temperature (b), and symptom duration (c) between the different BMI groups.

The severity of COVID-19 parameters was compared between the four groups of BMI, and significant results were found with regard to body temperature, SpO_2_, and symptom duration (Figure 3). The multiple regression analysis revealed that BMI positively predicted the body temperature and negatively predicted SpO_2_ (standardized *β* of −0.28 and 0.06; *P* = 0.02 and 0.001, respectively).

Regarding sex, when female patients were compared to male patients, a higher symptom score (25 vs 24, respectively; *P* = 0.04), higher mean sequelae (0.23 vs 0.17, respectively; *P* = 0.003), and longer symptom duration (15 vs 14 days, respectively; *P* = 0.04) were recorded.

**Figure 4:**
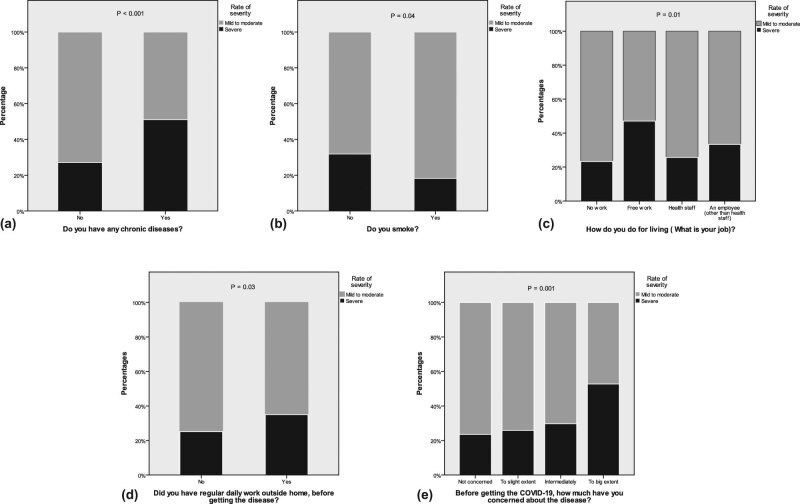
Comparison of the disease severity rating between (a) patients with and without comorbidities, (b) smokers and non-smokers, (c) patients with different jobs (d) patients with the presence or absence of regular daily work outside the home, and (e) patients with different degrees of concern about COVID-19.

When the presence of comorbidities was analysed, a significantly higher severity rating, symptom score, and body temperature and a lower level of SpO_2_ were found in the patients with a chronic disease compared to the patients without such a history (severity rating [4 vs 3], symptom severity score [27 vs 24], body temperature [38.9 vs 38.0], and SpO_2_ [90.5 vs 94.0], respectively; *P* < 0.05). About 51.0% of the patients with comorbidities was within the group of severe symptoms compared to 27.1% of the patients without comorbidities (*P* < 0.001; Figure 4a). Presence of comorbidities positively predicted the symptom score (standardized *β* of 0.18; *P* = 0.001).

**Figure 5:**
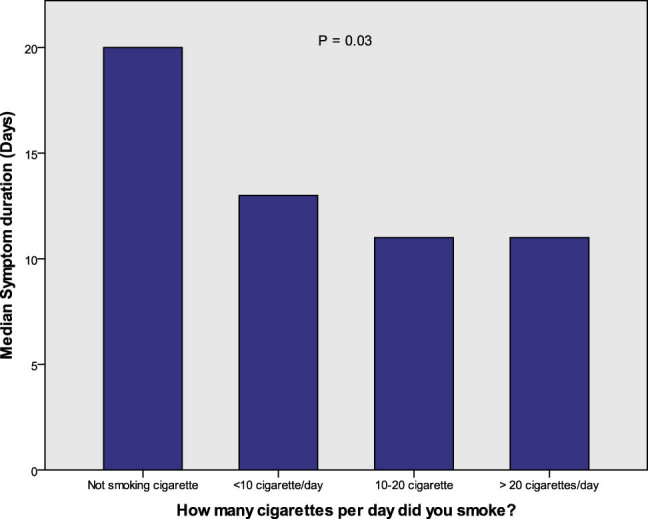
Comparison of the symptom duration between groups with different amounts of cigarette smoking.

Regarding smoking, when the severity parameters were compared between cigarette smokers and non-cigarette smokers, a significantly lower severity rating (2.48 vs 3.09, respectively, *P* = 0.01), symptom score (18 vs 24, respectively, *P* = 0.03), body temperature (37 vs 38°C, respectively, *P* = 0.045), and symptom duration (11.5 vs 14.0, respectively, *P* = 0.01) were revealed. However, no significant differences were observed between hookah smokers and non-hookah smokers. Regarding the SpO_2_ level in relation to smoking, no significant results were found. In assessing the amount of cigarette smoking, it was observed that the more cigarettes an individual smoked, the shorter their symptom duration (*P* = 0.03; Figure 5). When the severity rating was compared between the smoker and non-smoker groups, a lower percentage of smokers were in the severe group compared to non-smokers (18.2 vs 31.8%, respectively; *P* = 0.04; Figure 4b).

No significant difference was found in the COVID-19 severity parameters between alcohol drinkers and non-drinkers and between participants who regularly exercised and those who did not (*P* > 0.05).

Regarding the place of residence, the only parameter with a significant difference between the groups was the SpO_2_ level. The place of living significantly predicted the SpO_2_ level. The lowest SpO_2_ level was reported in the patients who lived in suburban areas compared to those who lived in urban areas in both the normal and overweight groups (suburban [87.0 vs 95.0] and urban [87.0 vs 93.5], respectively; *P* < 0.001). The number of family members and housing condition and the presence or absence of close contact had no significant relation with COVID-19 severity parameters (*P* > 0.05).

Regarding job and times spent out, the patients who were self-employed had significantly more severe disease compared to the rest, while those who were unemployed had the least severe disease (free work [47.1%] and no work [23.3%]; *P* = 0.01; Figure 4c). Regarding the analysis of the presence or absence of regular daily work to the severity rate, the severe group had a higher percentage of patients who had regular daily work outside of their homes compared to those without regular daily work (35.0 vs 25.1%; *P* = 0.03; Figure 4d). Meanwhile, multiple regression analysis failed to show the different types of job as a predictor of COVID-19 severity.

Concerns about COVID-19 positively predicted the symptom score (standardized *β* of 0.13; *P* = 0.01) and negatively predicted the SpO_2_ (standardized *β* of −0.16; *P* = 0.02). Patients who were more concerned about COVID-19 before contracting the disease had a higher mean symptom score, severity rating, and sequelae rate compared to those who were less concerned. The values were as follows for the patients who were not concerned, somewhat concerned, moderately concerned, and greatly concerned: symptom score = 22.1, 24.0, 26.0, and 26.9, respectively (*P* = 0.02); severity rating = 2.75, 2.92, 3.23, and 3.53, respectively (*P* < 0.001); and sequelae rate = 0.78, 0.92, 1.28, and 1.38, respectively (*P* < 0.001). A higher percentage of those who were greatly concerned had severe symptoms compared to those who were less concerned (52.7 vs 23.5%, respectively; *P* = 0.001; Figure 4e).

In regard to the protective measures, none of the protective measures had a significant correlation with the COVID-19 severity parameters (*P* > 0.05). The comparison in the frequency of protective measure use between mild-moderate and severe groups was not significant (Table S1).

### 3.6 Dietary supplements and COVID-19 severity

Among the studied participants, 165 (38.6%) individuals used prophylactic dietary supplements before contracting COVID-19. The percentage of use, the dosage of each dietary supplement (i.e. vitamin D, vitamin C, and zinc), and the duration are summarized in Tables 5 and 6. By multiple regression analysis, it was demonstrated that prophylactic vitamin D use was a significant predictor of COVID-19 severity; it negatively predicted COVID-19 severity (standardized *β* of −0.16; *P* = 0.03; Table 4).

**Figure 6:**
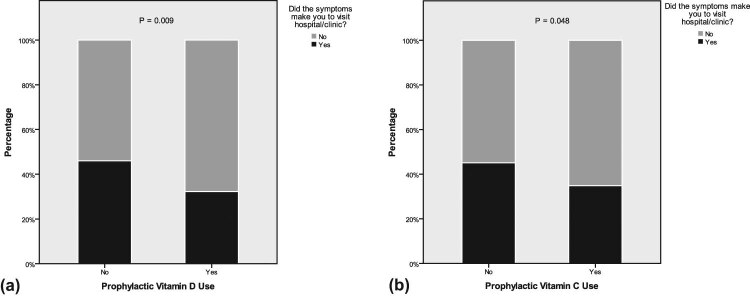
The proportion of hospital visit in patients with or without the use of prophylactic vitamin D (a) and vitamin C (b).

The proportion of hospital visits for patients who received prophylactic vitamins C and D supplements was statistically lower in comparison with patients who did not receive supplements (vitamin D [32.3 vs 46.0%] and vitamin C [34.8 vs 45.1%], respectively; *P* < 0.05; Figure 6).

The effect of the prophylactic dietary supplements on sleep disturbances was observed according to their daily dosage before COVID-19 infection. Less proportion of sleep disturbances was observed in patients who received >1,000 mg prophylactic vitamin C compared to patients who received smaller doses or patients who did not receive it (25, 43, and 40.5% in those with daily vitamin C dose of >1,000 mg, 500 mg, and none, respectively; *P* = 0.04).

Among the patients who received prophylactic vitamins C and D for more than 2 weeks, less proportion of hospital visit and a lower proportion of sleep disturbances were observed than patients who had been given a shorter duration of these supplements. Proportion of hospital visits in patients using vitamin C or D, for more than 2 weeks versus less than 1 week was as follow: vitamin C: 30.0 vs 56.5% (*P* = 0.03) and vitamin D: 30.4 vs 40.0% (*P* = 0.02). Frequency of sleep disturbances in patients using vitamin C or D, for more than 2 weeks versus less than 1 week was as follows: vitamin C: 40.0 vs 52.2% (*P* = 0.04), vitamin D: 39.1 vs 40.9% (*P* = 0.051), and zinc: 34.0 vs 41.4% (*P* = 0.02), respectively.

No significant difference was observed between the patients with and without prophylactic use of zinc and vitamins C and D in terms of the hospitalization, use of medication, use of plasma, or use of oxygen therapy (*P* > 0.05).

**Table 5:** Rate of receiving prophylactic dietary supplements and daily dosage in the studied participants

Dietary supplement use	Total	
	Yes	No
	*N* (%)	*N* (%)
**Dietary supplements (any)**	165 (38.6)	263 (61.4)
**Vitamin D**	127 (29.7)	300 (70.1)
Daily dosage		
<1,000 IU	39 (30.7)	
1,000–4,000 IU	49 (38.6)	
>4,000 IU	28 (22)	
Not known	11 (8.7)	
**Vitamin C**	132 (30.8)	295 (68.9)
Daily dosage		
500 mg	81 (61.4)	
1,000 mg	36 (27.3)	
>1,000 mg	4 (3)	
Not known	11 (8.3)	
**Zinc**	111 (25.9)	317 (74.1)
Daily dosage		
<50 mg	58 (52.3)	
50–100 mg	48 (43.2)	
Not known	5 (4.5)	

**Table 6:** Duration of use of prophylactic dietary supplements based on the participants studied

Duration	Vitamin D	Vitamin C	Zinc
	*N* (%)	*N* (%)	*N* (%)
<1 week	22 (5.2)	23 (5.4)	29 (6.8)
1–2 weeks	36 (8.4)	49 (11.5)	35 (8.2)
>2 weeks	69 (16.2)	60 (14.1)	47 (11)
Not using it	37 (8.7)	32 (7.5)	53 (12.4)
None	263 (61.6)	263 (61.6)	263 (1.6)

## 4 Discussion

In this study, nearly 94.9% of patients experienced varied symptoms of the disease. This result is in agreement with a study conducted in South Korea in which the majority of COVID-19 cases diagnosed by surveillance testing were symptomatic [54]. Regarding the symptom profile, the most common symptoms were malaise, myalgia, headache, fever and/or rigour, and sweating and were experienced by more than 90.0% of the patients, followed by cough, loss of taste and/or smell, and sore throat in about three-fourths of the patients. The occurrence of these symptoms in patients with COVID-19 has also been reported elsewhere [5,53,55]. In a study on the outpatient US population in a multistate healthcare system network from March to June 2020, malaise was found to be the most common symptom, followed by cough and headache [56]. In our study, regarding the general severity rating, 70% of the patients showed mild-moderate symptoms, while 30% exhibited severe symptoms. About 96.6% of the symptomatic patients reported fever, cough, or SOB as typical symptoms, and these three typical symptoms presented in more than half of these patients. This was also found by CDC surveillance from January to April 2020 for symptom profiles among a convenience study sample of COVID-19 patients in the United States [53]. A large proportion (about three-fourths) of the studied patients presented with GIT symptoms. The presence of at least one GIT symptom was also recorded in most previous studies [53,57,58,59].

In the current study, significant differences in severity parameters were notable in regard to age (the age group >65 years showed more severe disease). The association of age with severity has been shown in previous studies [56,60].

The median symptom duration in our survey was 14 days; in other studies, a median symptom duration of 13–16.5 days was reported [56,60]. In the current study, malaise remained in about half of the participants; in a previous study, malaise/fatigue and cough were reported as the symptoms that remained in a majority of outpatients with COVID-19 [56].

In our study, the lowest level of SpO_2_ was recorded in the obese patients. This correlation could be explained by the tendency of obese people to develop metabolic syndrome [61], which could interfere with their metabolic response during their COVID-19 infection and the effect of excess fat deposition on lung function [62,63,64], the obese also have lower vitamin D level [65]. The lowest reported SpO_2_ was recorded in patients over 65 years old. This result is in line with the findings of another study in which the researchers found that patients with SpO_2_ values of 90.0% or less were older [66]. We found that the SpO_2_ level was not correlated with sex.

In our study, we noted that those who had one or more comorbidities suffered a more severe form of COVID-19 in comparison with those who did not. This finding is in parallel with other studies that showed that the clinical manifestations of COVID-19 in those with underlying chronic illnesses were severe [67,68,69,70].

Moreover, only a minority (1/7) of the participants were smokers, and the reason could be greater proportion of female patients in our study, as smoking was found to be less prevalent in women in our community [71]. The smoker patients had a significantly lower severity rating compared to non-smokers; the symptom duration was longer in patients who smoked fewer than ten cigarettes per day in comparison with those who smoked more than 20 cigarettes per day. These results are in concordance with a large observational study in which smokers were less at risk of contracting COVID-19. Although the reason for this is unknown, the pathophysiology of the disease at the cellular level may play a role [72]. Furthermore, a meta-analysis of five studies on active smoking did not find an association between smoking and disease severity [10], while other studies showed that cigarette smokers developed more severe infections and worse outcomes in comparison with non-smokers [11,12,14]. A systematic review suggested that cigarette smoking can increase the risk of COVID-19 infection as well as the severity and mortality rate among hospitalized patients [9].

The lower severity rating among smokers was observed since the majority of our participants were outpatients in comparison with the hospitalized COVID-19 patients in the aforementioned study [9]. Among hospitalized COVID-19 patients, worse outcomes were found among smokers compared to non-smokers. The same could be said for a large observational study in Israel in which one-third of the hospitalized patients had a moderate to severe infection [72]. Smoking is not included in the global COVID-19 risk factor guidelines [13].

In the current study, lower SpO_2_ levels were recorded among hookah smokers compared to cigarette smokers. A record of the SpO_2_ levels among different types of smokers could not be found, but the use of hookah among younger adults and the sharing of the mouthpiece between them could increase the risk of SARS‐CoV‐2 transmission [15]. Alcohol consumption in our study did not relate to COVID-19 severity.

In the present study, concern about COVID-19 in two-fifths of the patients was to a moderate-high extent, which is in line with a local study in which about half of the Kurdish population consider COVID-19 to be a major harm to their lives [73], which reflects a study on the US population [21]. The patients who were concerned about COVID-19 before contracting the disease were mostly those with comorbidities who might have been more affected by the news from social media and television about worse COVID-19 outcomes in patients with chronic diseases. In addition, a strong association between stress/concern about COVID-19 and symptom severity was found in this study. Higher rates of COVID-19 infection, severity, progression, and complication among patients with previous mental illness were observed [74]. Moreover, a known negative impact of stress was observed on immune cells/functions [75] and disease progression due to alterations in the circadian rhythm [76]. No association was observed between exercise and COVID-19 severity in the present study, and the impact of exercise on COVID-19 severity has not yet been studied.

In our study, the disease severity was analysed among patients with different residency locations and housing condition: The lower SpO_2_ level observed in our suburban study participants could have been the result of having less access to health facilities and services. Another possibility could be poorer housing conditions and the greater number of households in the suburban areas compared to urban areas; thus, a lower capacity for of social distancing. In the current study, no significant correlation was found between COVID-19 severity and housing conditions or number of family members/household. In contrast, other studies have shown that areas with a higher proportion of poor housing conditions are associated with a higher rate of infection and COVID-19-related mortality [27,28]. Moreover, the participant’s job did not predict the severity of COVID-19.

In this study, a large proportion (around two-thirds) of the patients did not apply social distancing from known or suspected patients with COVID-19. According to a systematic review, a physical distance of more than 1 m offers more protection in comparison to a distance of less than 1 m; and it is important to decrease the adverse outcomes, morbidity, and mortality of COVID-19 [34]. In the current study, no significant association was found between the severity rating and the use of protective measures. At the time of writing this article, no population-based study was conducted on the effectiveness of face mask wearing[77].

Prophylactic use of vitamins C and D and zinc was observed in less than one-third of the participants. Among the three micronutrients, prophylactic vitamin D was significantly associated with less severity. The duration of taking prophylactic micronutrients varied, and less than half of the prophylactic supplement users had taken them for over 2 weeks before contracting COVID-19. Hospital visits and sleep disturbances among participants who had taken prophylactic vitamins C or D for longer than 2 weeks were significantly less recorded compared to the participants who took these vitamins for less than 2 weeks. Moreover, regardless of the duration of using these supplements, fewer participants taking prophylactic vitamins C and D had visited hospital compared to those not taking the supplements. Significant correlation of vitamin D with COVID-19 severity and other results supports their probable prophylactic benefits during the COVID-19 pandemic [39,40,43,44,78,79,80,81], suggesting the need for initial high-dose vitamin D intake, since a routine daily dose of vitamin D requires several weeks to normalize the vitamin level to be able to boost immunity against COVID-19 infection [39,40]. Moreover, a previous study found that the vitamin C level is greatly reduced in critically ill patients, necessitating appropriate supplementation [82].

Although there is no consistency regarding the dose of these supplements in the prevention and treatment of COVID-19 [47], we found that the patients who took more than 1,000 mg daily of prophylactic vitamin C had a significantly lower proportion of sleep disturbances than the patients who took less than this daily dose. To support the optimum dose of these supplements, further studies are required.

### 4.1 Strengths and limitations of the study

The strengths of this study are that a patient-based study of COVID-19 patients without critical condition and ICU admissions are scant. This group of patients has been ignored in the literature even though they comprise a majority of COVID-19 patients. Most of these patients do not need hospitalization and the management and care of their COVID-19 infection are done at home. Thus, the study of the severity, morbidity, and symptom profile of these patients is of great importance, as are the frequency of their use of protective measures and prophylactic supplements and the rate at which they seek medical assistance. Furthermore, our information about the correlation of COVID-19 symptom severity with patients’ socio-demographic characteristics and prophylactic dietary supplement use will be current at the time of publication.

However, this study also has limitations. As it is a patient-based survey, the most severe patients who required ICU could not be included, and the data are more subjective than objective and affected by the personal perspectives, feelings, and ratings of severity of patients. There could have been variations in their pain thresholds or exaggerations in their symptoms and severity, and recalling in their concern about the disease before getting infected with it, which would affect their responses. Another shortcoming could have resulted from the patients’ recall of their use of prophylactic supplements before their infection and their symptoms and the severity during the infection, as the data were collected after complete recovery from COVID-19. Although the medication, dosage, and duration options from which the patients chose were written out clearly, reporting the drug name, dosage, and duration of use could be difficult for some people among the general population and be subject to mistakes.

Another limitation of this study is that the convenience sampling of the relatively small sample size did not allow for proper analysis of several parameters such as smoking condition, place of residency, and dose of supplementary medication used, especially after stratification by age and BMI. Another limitation is that few elderly and paediatric or underweight patients participated in this study. Otherwise, we could have found more reliable outcomes of COVID-19 in these groups and related them to COVID-19 severity parameters. Moreover, as it is a patient-based survey and the data were collected from outpatients, not all the patients were RT-PCR confirmed COVID-19; some patients were diagnosed based on typical HRCT findings reported by a radiologists; and a few of them based on typical symptoms, their contact history in combination with CXR finding, and/or serology by specialized physician. However, for the same purpose, serum 25-hydroxyvitamin D level could not be measured for the patients.

## 5 Conclusions and recommendations

In this study, we concluded that several factors are associated with increased risk of COVID-19 severity. Stress, older age, and comorbidities were the most common and critical risk factors. The use of protective measures has no significant association with disease severity. Vitamin D is significantly associated with a lower severity rating. Furthermore, according to this study, exercise was not associated with COVID-19 severity, while cigarette smoking, but not hookah smoking, was associated with less severe infection among COVID-19 outpatients.

As stress is among the risk factors for COVID-19 severity, the efforts by the authorities to help calm people and minimize panic spreading by social media are recommended in line with stress management.

The use of prophylactic vitamin D is suggested before and during COVID-19 infection. Although cigarette smoking is associated with less severe COVID-19 among outpatients, it is not encouraged to smoke or to increase smoking during the COVID-19 pandemic, as smoker patients who were hospitalized showed a higher risk of ICU admission and ventilator use with worse ARDS.

Overall, a large population-based study is recommended to relate the symptom severity in COVID-19 patients to socio-demographics and prophylactic dietary supplements and to compare patients with healthy individuals to relate these parameters with the rate of COVID-19 infection.

## Figures and Tables

**Figure A1:**
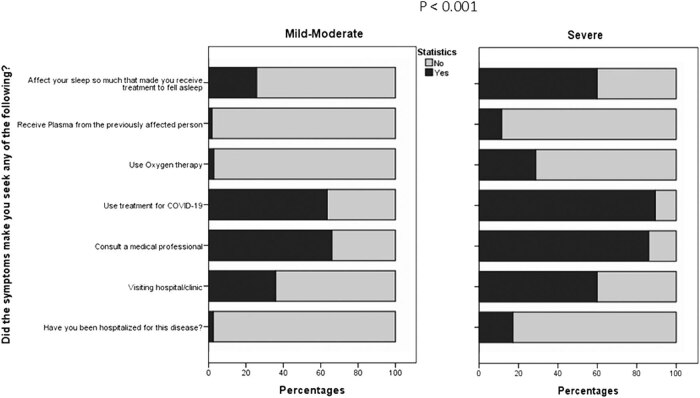
Comparison of the severity parameter-II data in between the mild-moderate and severe groups.

## Appendix

**Table A1:** The frequency of protective measure use before contracting the disease between patients with mild–moderate and severe infection

Protective measure use	Total symptomatic *N* = 407			Protective measure use		
	No	Yes	Protective measure use in patients with different rate of severity	No	Yes	*P*-value
	*N* (%)	*N* (%)		*N* (%)	*N* (%)	
Facial mask	139 (34.2)	268 (65.8)	Mild-moderate	90 (64.7)	195 (72.8)	0.06
			Severe	49 (35.3)	73 (27.2)	
Social distance	277 (68.06)	130 (31.9)	Mild-moderate	189 (68.2)	96 (73.8)	0.25
			Severe	88 (31.8)	34 (26.2)	
Hand wash/Disinfectant	106 (26.04)	301 (73.9)	Mild-moderate	82 (77.4)	203 (67.4)	0.06
			Severe	24 (22.6)	98 (32.6)	
Stay away from contact	237 (58.2)	170 (41.8)	Mild-moderate	168 (70.9)	117 (68.8)	0.65
			Severe	69 (29.1)	53 (31.2)	
